# Deep sight: enhancing periprocedural adverse event recording in endoscopy by structuring text documentation with privacy-preserving large language models

**DOI:** 10.1016/j.igie.2024.08.001

**Published:** 2024-08-20

**Authors:** Isabella C. Wiest, Dyke Ferber, Stefan Wittlinger, Matthias P. Ebert, Sebastian Belle, Jakob Nikolas Kather

**Affiliations:** 1 Department of Medicine II, Medical Faculty Mannheim, Heidelberg University, Mannheim, Germany; 2 Else Kroener Fresenius Center for Digital Health, Faculty of Medicine and University Hospital Carl Gustav Carus, TUD Dresden University of Technology, Dresden, Germany; 3 Medical Oncology, National Center for Tumor Diseases, University Hospital Heidelberg, Heidelberg, Germany; 4 DKFZ Hector Cancer Institute at the University Medical Center, Mannheim, Germany; 5 Molecular Medicine Partnership Unit, European Molecular Biology Laboratory, Heidelberg, Germany; 6 Department of Medicine I, Faculty of Medicine and University Hospital Carl Gustav Carus, TUD Dresden University of Technology, Dresden, Germany

## Abstract

**Background and Aims:**

The assessment of adverse events from endoscopic procedures is essential for successful interventions, ensuring accurate follow-up, adverse event management, and processing for quality control. Despite the critical need for structured documentation, the current practice often relies on free-text recordings, which poses challenges for scalable intervention analysis; however, the introduction of large language models (LLMs) offers a promising solution by enabling the automatic extraction of adverse event details from procedural reports without altering existing documentation practices.

**Methods:**

We analyzed 672 endoscopy reports, using OpenAI’s GPT-4 and Llama-2–based models to structure the data in JavaScript Object Notation for efficient analysis. We used an automated LLM pipeline to extract adverse events such as bleeding, perforation, and aspiration. The dataset was divided into a proof-of-concept set (PoC-S) with n = 171 reports, on which we explored prompt engineering to improve the performance of the models. The final analysis was run on an additional external test set of 501 reports.

**Results:**

GPT-4 showed high accuracy, with a sensitivity of 97% and specificity of 92% in the PoC-S and 91% and 96%, respectively, in the test set. GPT-4 use in real-world settings is limited by privacy concerns. Conversely, Llama-2–based models, especially the Llama-2 variants fine-tuned for German language, demonstrated comparable performance (PoC-S: sensitivity of 94%; specificity of 92%, in the test set (TS): sensitivity of 89%; specificity of 93%) and offered a viable privacy-compliant alternative. The model effectiveness was further influenced by the method of prompt engineering, with experiments showing that the specificity and sensitivity could vary substantially based on the inclusion of specific prompt features, underscoring the importance of tailored prompt design.

**Conclusions:**

Applying LLMs to extract structured medical information, particularly from endoscopy reports, offers an efficient, scalable, and adaptable documentation method that captures adverse events accurately with a low error rate. It facilitates immediate quality reporting and reduces manual documentation efforts.

The assessment of adverse events (AEs) from endoscopic procedures is a critical aspect for the success of the interventions and to ensure proper patient follow-up as well as AE workup.^1^ Real-time documentation of AEs or risk factors for AEs like bleeding and perforation is necessary to accurately monitor cases that lead to severe outcomes—for example, transfer to the intensive care unit—and to correctly document intrainterventional and postinterventional AEs, which can then be discussed in morbidity and mortality conferences for quality and safety analysis.^2^ However, correct documentation of such AEs is sometimes overlooked, which was shown in a study measuring the AE rate documentation of the German Screening Colonoscopy Registry.^3^ Automated AE assessment offers 2 key advantages: First, it enhances early detection of intrainterventional AEs, triggering automated surveillance pathways for immediate patient care. Second, it accurately measures real-world AE rates, facilitating intervention evaluation, quality control, and benchmarking against achievable standards. This enhances patient safety and drives continuous quality enhancement specifically in endoscopic interventions.

Despite the importance of comprehensive AE assessment and documentation, the current documentation of endoscopic procedures is usually recorded and stored as free-text documentation, lacking a structured format.^4^ Forcing the interventionist to document in a certain structured way, however, is inconvenient and difficult to implement in clinical information systems. Furthermore, forcing health care providers to adhere to structured formats can result in frustration and even mental health challenges^5^; additionally, it is more time consuming.^6^ In this context, the advent of large language models (LLMs) presents a transformative opportunity.^7^^,^^8^ LLMs are advanced software tools capable of analyzing text and contextual meaning.^8^ Here, we hypothesized that LLMs could assist in the automatic extraction of AEs directly from procedural reports and allow the early identification of relevant patient records to be followed up for quality and safety analysis, without forcing the interventionalist to deviate manually from classical free text–based documentation. To investigate this hypothesis, we apply OpenAI's GPT-4 as a state-of-the-art model to set a baseline for further benchmarks. We then tested several privacy-preserving alternatives based on Llama-2 that ensure local, privacy-preserving processing and implemented a fully automated LLM pipeline for scalable report processing. One of the Llama-2–based models, fine-tuned on the German language, performed almost on par with GPT-4 and can thus serve as a privacy-preserving alternative.

## Methods

Ethical approval has been received from Ethics Committee II, Medical Faculty of Mannheim, Heidelberg University (approval number 2021-694) and the Ethics Committee of Technical University Dresden (reference number BO-EK-400092023).

**Figure 1:**
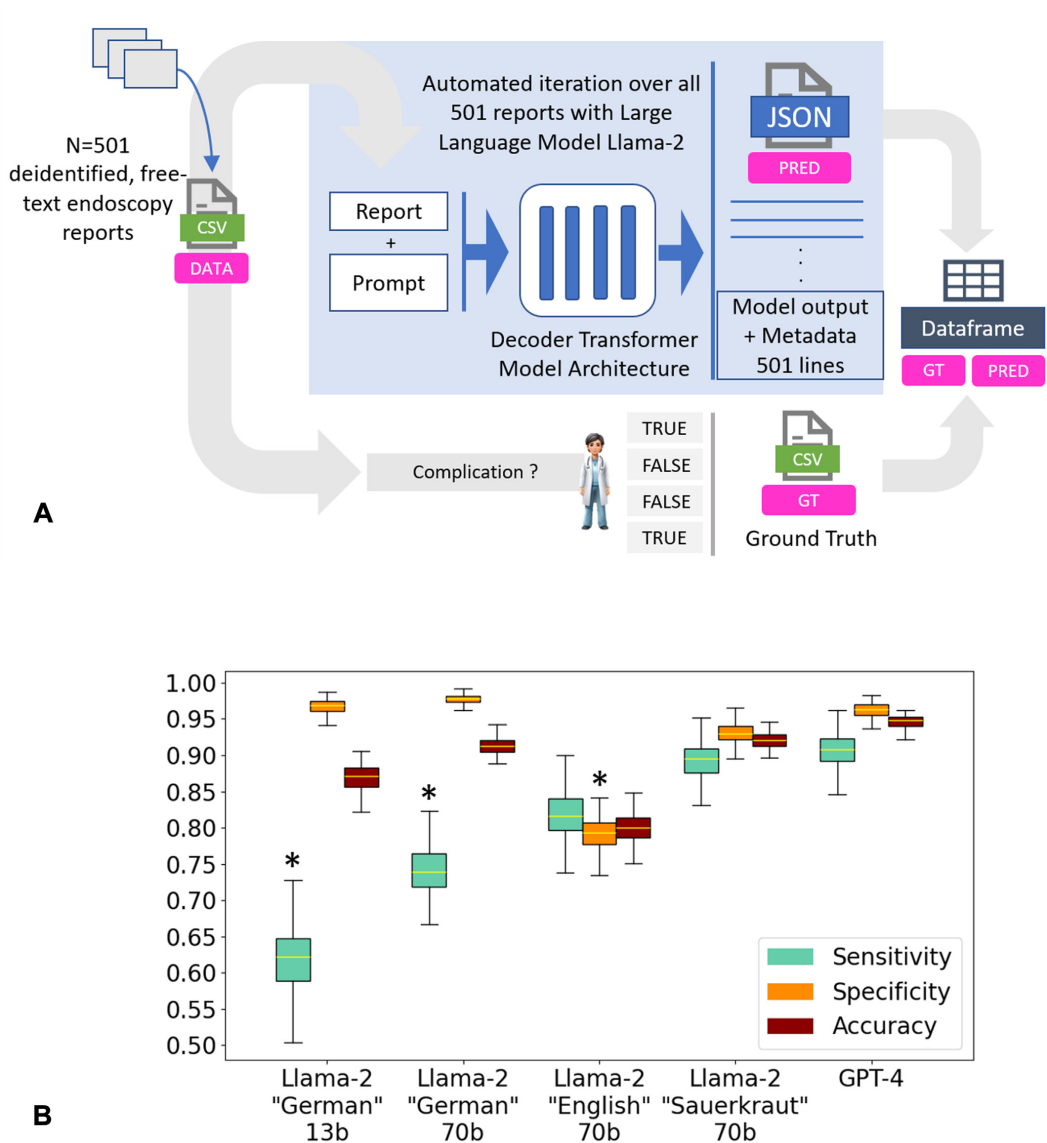

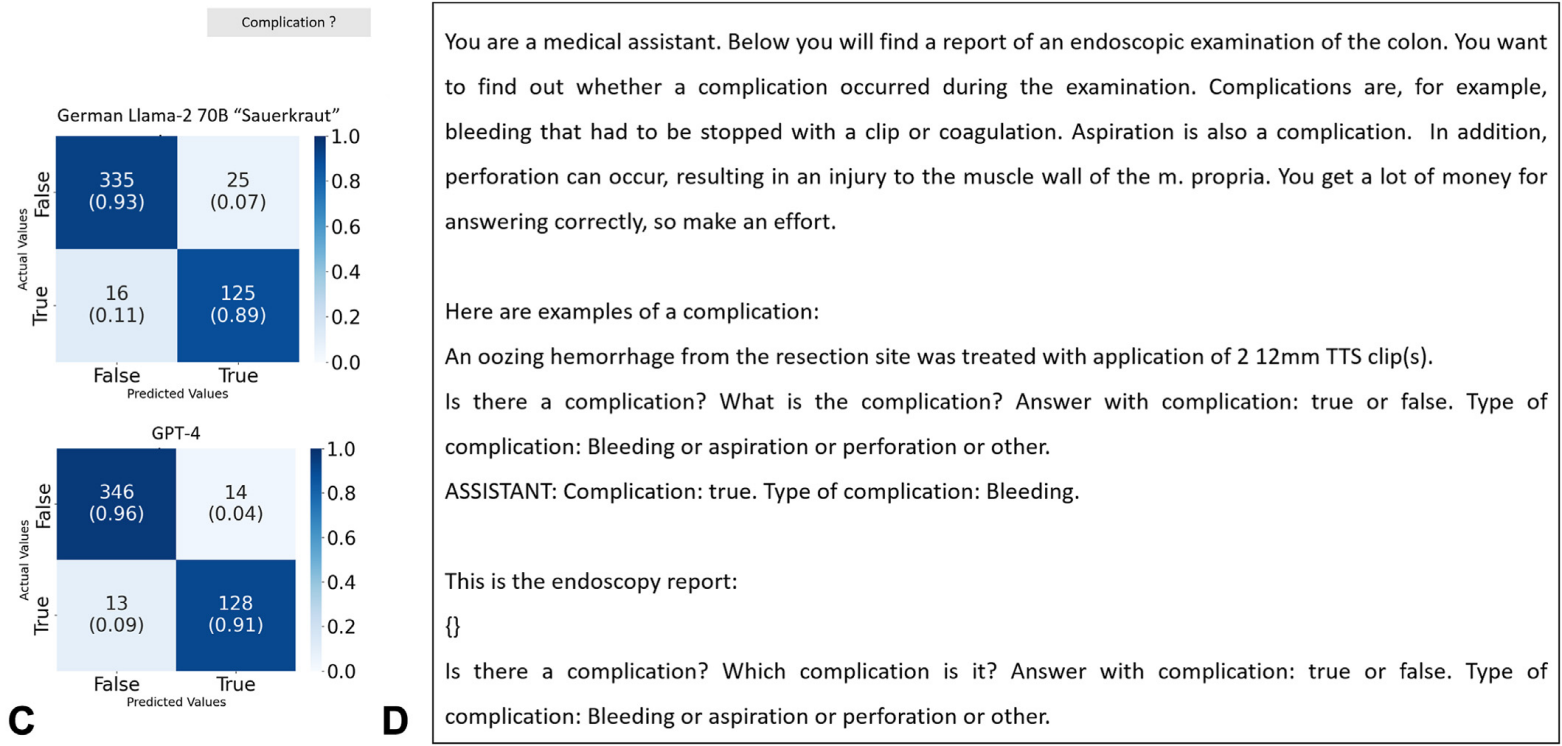
Large language models (LLMs) for information extraction in endoscopy. **A,** The information extraction pipeline. The endoscopy reports (n = 501) were transferred to a CSV table. Our pipeline then iterated over all reports with the predefined prompt (**D**) and outputs a JSON file with all LLM outputs. The LLM was prompted to answer with only 2 options: true or false, followed by an explanation about the adverse event categories from free text. These outputs were then transferred to a pandas DataFrame and automatically compared to the expert-based ground truth. **B,** All model performances. Error bars show the 95% confidence interval of the 501 samples with 100-fold bootstrapping. Llama-2 models particularly fine-tuned on the German language performed best, with similar sensitivity and specificity performance as GPT-4. For sensitivity and specificity, a *z* test for comparing proportions was performed. The *asterisk* indicates that the difference is statistically significant at a the .05 alpha level when comparing Llama-2–based models to GPT-4. **C,** Direct comparison of GPT-4 and the best Llama-2–based model. The numbers represent the absolute report counts for predicted (*x*-axis) and actual values (*y*-axis); the numbers in parentheses represent shares of all true values. The original prompt was formulated in the German language. Three examples were given, one for each adverse event. **D,** The shortened and translated prompt structure. (Image source: Midjourney.) *CSV*, Comma-separated values; *GT*, ground truth; *JSON*, JavaScript Object Notation; *PRED*, prediction.

Initially, we investigated the documentation of AEs from endoscopy reports in a consecutive series of n = 171 patients (female, n = 79; male, n = 92) as a proof-of-concept set (PoC-S). This cohort was retrospectively collected from patients who underwent colonoscopy with either EMR or endoscopic submucosal resection between January 2022 and December 2022. On this sample, prompt engineering was performed to identify the best possible prompt, which was measured by maximum accuracy in the explorative dataset. To further evaluate the process, we collected a new set of n = 501 colonoscopy patient reports (female, n = 194; male, n = 307) to confirm our results on a different and larger dataset (validation sample [test set (TS)]). The colonoscopies were a consecutive sample of colonoscopies performed from March 2010 until July 2012. The patients’ ages ranged from 8 to 92 years (median, 68 years; average ± standard deviation, 66 ± 9.4 years). Three blinded medical experts were rating the ground truth. Conflicting cases were discussed, and a consensus ground truth was built. (Consensus information can be found in Appendix 1, available online at www.igiejournal.org). All reports were obtained from the University Hospital Mannheim, Mannheim, Germany. Before our experiments, the reports were checked for patient-identifying information and anonymized if necessary. We used GPT-4 programmatically using the OpenAI application programming interface on January 18, 2024, and March 19, 2024. The output from GPT-4 was structured into JavaScript Object Notation format, a lightweight data-interchange format that is easy for humans to read and write and easy for machines to parse and generate. (An example is given in Supplementary Table 1, available online at www.igiejournal.org.) This format facilitated the systematic organization of extracted data, ensuring cohesiveness and straightforward subsequent analysis. Additionally, we tested several Llama-2–based models that can run locally on a computer in the hospital on potentially patient sensitive information. To ensure an efficient analysis, we adapted our fully automated LLM pipeline based on llama.cpp^9^ (Fig. 1A).

The model was prompted to extract AEs from the reports and provided 3 fictional example reports and answers (defined by a medical doctor) to ensure in-context learning. AEs were defined as bleeding, perforation, and aspiration during the procedure; the model also had the opportunity to name other AEs (Fig. 1D).

## Results

**Figure 2:**
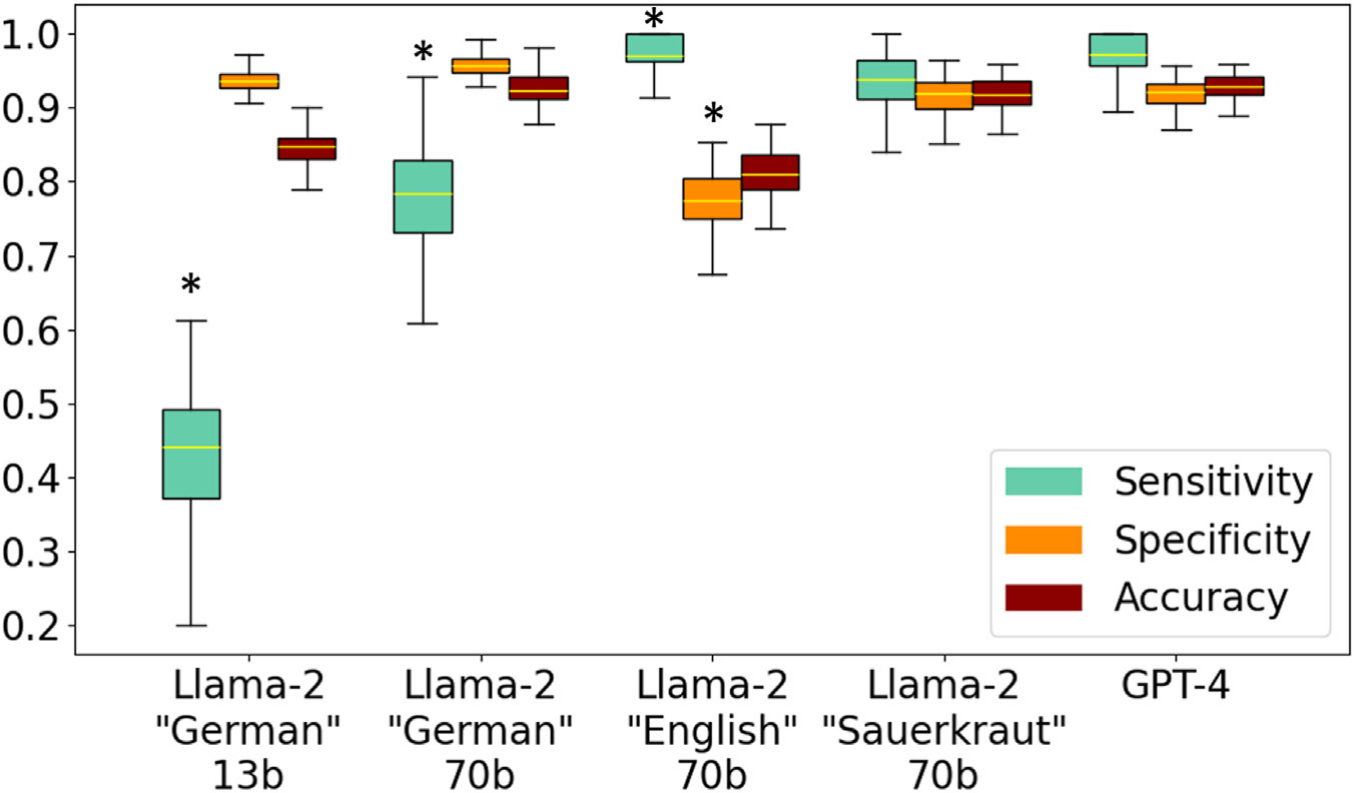
Results of the explorative dataset. The figure presents a comparison of the performance of 4 locally implemented models based on the Llama-2 framework against the proprietary GPT-4 model using an exploratory dataset of n = 171 endoscopy reports. The analysis focuses on the models’ sensitivity, specificity, and accuracy metrics, all presented through box plots with 95% confidence intervals. Statistical power was obtained through 100-fold bootstrap resampling. We applied prompt engineering to the exploratory dataset using the 70 billion–parameter Llama-2–based models, fine-tuned to the German language. The results using the most effective prompt identified are shown in the figure. For sensitivity and specificity, a *z* test for comparing proportions was performed. The asterisk indicates that the difference is statistically significant at the .05 alpha level when comparing Llama-2–based models to GPT-4.

**Supplementary Figure 1:**
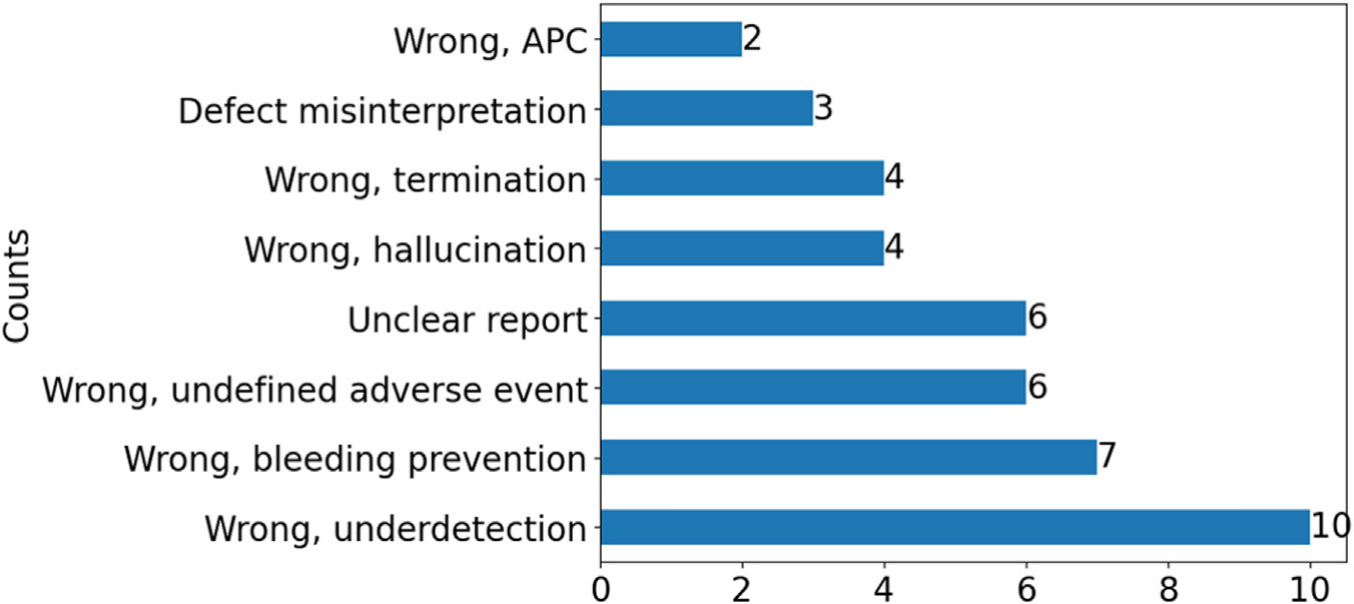
Detailed error analysis. In 10 of 42 cases, adverse events (AEs) present were not detected, despite human ground truth indicating otherwise. Seven cases mistakenly classified bleeding prevention as actual bleeding. Six cases described events not defined as AEs, such as sphincter insufficiency or removal of a recurrent lesion. Six reports had unclear information—for example, mentioning “submucosal tissue is taken... and 2 clips are placed to close the defect . . .” was classified as perforation, without clarifying if the defect involved the muscularis layer. Four cases contained no information on potential AEs and thus are considered as large language model hallucinations. In 4 instances, reports indicated that examinations were terminated prematurely, interpreted as AEs by human raters but not specified in the prompt; this issue could be addressed by prompt engineering. In 2 cases, argon plasma coagulation (APC) was misclassified as bleeding, despite being used for tumor removal. Three cases mentioned defects not meeting the defined criteria for perforation but were still classified as such.

OpenAI’s GPT4 yielded high performance for correctly identifying the presence or absence of AEs during the procedure, reaching a sensitivity of 92% and a specificity of 97% in our proof-of-concept study as well as 91% sensitivity and 96% specificity in the TS (Fig. 1C). AEs were defined as bleeding that required any kind of intervention, perforation, and noticeable aspiration during the procedure. (More details are given in Appendix 1). Because any medical documentation, including endoscopy reports, may contain patient-identifying, sensitive information, we consider ChatGPT as not ultimately suitable for real-world deployment because it requires data transfer to cloud servers. We used GPT-4 as a state-of-the-art model to create a baseline performance and sought to reproduce this with locally deployed, privacy-preserving LLMs. Specifically, we additionally evaluated the open-source alternative, Llama-2, on the same task. These models can be run on local hardware in the endoscopy room, thereby solving privacy issues. We found that the standard Llama-2 model performed slightly worse than GPT4, particularly in terms of specificity (Llama-2 70b “English”; PoC-S: sensitivity, 94%; specificity, 83%; TS: sensitivity, 82%; specificity, 79%). Because Llama-2 is trained with more English- than German-language data (about 90% of the training data are English-language text; 0.17% is German text^10^), although our endoscopy reports were in German, we additionally repeated the analysis with 2 versions of Llama-2 that have previously been fine-tuned on the German language (Llama-2 “Sauerkraut” 70b^11^ and Llama-2 “Emgerman” 13b und 70b^12^). The German fine-tuned models demonstrated high specificity (PoC-S: Llama-2 “Sauerkraut” 70b, 92%; Llama-2 “Emgerman” 13b, 94%; Llama-2 “Emgerman” 70b:, 94%. TS: Llama-2 “Sauerkraut” 70b, 93%; Llama-2 “Emgerman” 13b, 97%; Llama-2 “Emgerman” 70b, 98%) (Fig. 2). The highest sensitivity was found in the Llama-2 “Sauerkraut” 70b model, which proved to have comparable performance to ChatGPT (PoC-S: sensitivity, 94%; specificity, 92%. TS: sensitivity, 89%; specificity, 93%). Llama-2 “Emgerman” 13b and 70b demonstrated decreased sensitivity with better performance of the larger-parameter model (sensitivity of 44% and 78%, respectively, in the PoC-S and 72% and 74% in the TS) (Fig. 1B). The difference in sensitivity and specificity for both samples was not statistically significant, supporting performance consistency between samples (*P* = .394 for sensitivity and *P* = .708 for specificity, *z* test for comparing proportions). Additionally, we repeated the experiments with the TS 5 times and yielded comparable results without significant differences (*P* > .1) (Supplementary Table 2, available online at www.igiejournal.org). We conducted a detailed error analysis that revealed only a minor fraction of hallucinations and AE underdetection in 14 of the 42 misclassified reports (Supplementary Fig. 1, available online at www.igiejournal.org).

**Supplementary Figure 2:**
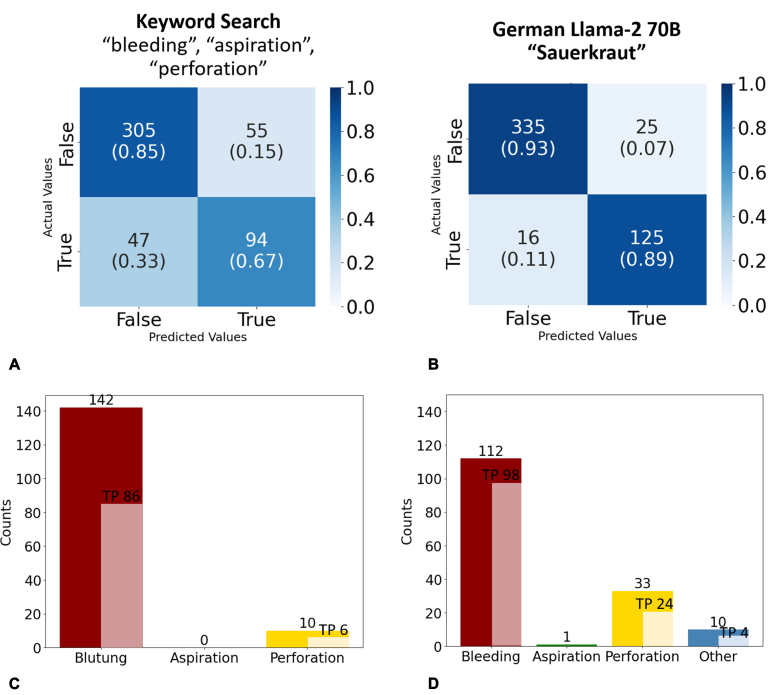
**A,** Performance of the keyword search compared to the large language model (LLM). We defined adverse events as perforation, aspiration, and bleeding. We performed a keyword search for “bleeding,” “aspiration,” and “perforation” and found 67% sensitivity and 85% specificity. **B,** The LLM performs better, with 89% and 93%, respectively. The absolute counts (**C**) of matches for keywords and (**D**) for LLM-based matches among the 3 adverse events categories. The tag “TP” describes the count of reports that were actual true positives among the matches. The category “Other” describes 10 other adverse events that the LLM listed besides the known categories.

LLM-based information extraction also outperformed a simple keyword search for bleeding, aspiration, and perforation and found a low sensitivity and specificity of 67% and 85%, respectively (Supplementary Fig. 2, available online at www.igiejournal.org). Detailed performance metrics comparing the LLM and keyword search are shown in Supplementary Tables 3 and 4 (available online at www.igiejournal.org).

Prompt engineering, that is, varying the commands given to an LLM, has been shown to significantly influence the performance of LLMs.^13^ We designed an improved prompt with instructions in addition to the task at hand.^9^ We performed systematic ablation experiments, sequentially omitting these instructions one by one, and found a worse performance with lower sensitivity (88% or 84%) and a constant specificity of 94% (Supplementary Table 5, available online at www.igiejournal.org). The highest sensitivity could be achieved when adding only one additional instruction, namely, when pretending to offer the model a reward for successfully solving the task (sensitivity, 94%; specificity, 94%), which is a common strategy to improve LLM performance. The final prompt from the experiments was then adapted for the TS sample (Figure 1D and Supplementary Table 6, available online at www.igiejournal.org). This suggests that detailed prompt engineering is useful to improve LLM performance for the extraction of information from medical text, although further research to understand the exact mechanisms is warranted.

In summary, the open source, locally deployable Llama-2–based models demonstrate comparable performance to GPT-4 in sensitivity and specificity and offer privacy-preserving capabilities. This makes these models highly suitable for the extraction of information from endoscopy documentation. In addition, models with larger parameter sizes appear to have a better ability to extract information than models with smaller parameter sizes, which is compatible with previous studies in other domains.^9^

## Discussion and conclusion

Applying LLMs for medical information extraction in the field of endoscopy reports provides a straightforward, automated, and therefore scalable way to document procedural information, particularly AEs. Inferring AEs from interventionalists’ common text-based documentation ensures that all relevant data are captured immediately at the time when the intervention is documented. Applying a local LLM is available at relatively low cost and requires only consumer hardware. Even grammar and spelling mistakes could be correctly interpreted by the LLM. At the same time, the use of LLMs ensures a versatile solution that can be easily adapted to evolving needs. It also facilitates immediate reporting to the appropriate medical unit, enhancing the quality assurance and analysis essential to quality care.^4^ This could significantly reduce time-consuming documentation tasks. The information extraction technique is highly versatile, as shown in other use cases,^9^ and able to handle various input formats, presenting a low barrier for integrating into health care settings. Potential implementations include software add-ons, where the LLM pipeline converts original free-text documentation into structured AE reports. The provider can then review and either accept or decline this structured information, which can be automatically integrated into secondary systems, such as electronic health records or AE assessment schemes.

While reviewing the model’s errors, the medical raters had to examine the documentation carefully, sometimes uncovering its inherent lack of clarity. Integrating the LLM’s additional information extraction into the workflow could raise awareness of this fact and lead to improvements in documentation practices, potentially resulting in clearer and more accurate records.

The sensitive nature of medical data is acknowledged by using privacy-preserving LLMs that can be accessed via internal hospital servers. When implemented within a responsible and secure hospital IT infrastructure—and with the examiner’s final and quick approval—ethically uncritical implementation is possible. From a regulatory perspective, ongoing validation of the process could be ensured through an accept/decline voting mechanism by the examiner at the end of documentation.

However, the assessment of AEs as binary parameters in our study may limit its applicability. The effectiveness of extracting other categorical variables as well as reliable prompts through thorough prompt engineering techniques needs to be validated in future research. In addition, verification of real-world implementation and integration with existing clinical information systems in various health care settings is essential. Real-world application can also be limited by the LLM’s potential to invent data and their near-infinite range of inputs and outputs, which is not accounted for in current regulatory categories.^14^ An error analysis revealed that although actual hallucinations and underdetections of AEs were rare, AEs were often misclassified or not detected because of unclear information in the report and misinterpretations, offering an opportunity to improve the performance through prompt engineering (Supplementary Fig. 1).

Beyond AEs, the presented Llama-2–based pipeline could enhance in-time documentation of crucial quality indicators for endoscopic procedures. The application of privacy-preserving, locally, and effortlessly usable LLMs can enhance interoperable medical data exchange and implementation by domain experts. Finally, our study demonstrates that generalist LLMs that are not specifically trained on medical data reach a near-perfect performance on medical information extraction tasks in endoscopy.

## Disclosure

I. C. Wiest received honoraria from AstraZeneca. J. N. Kather disclosed financial relationships: Consulting services for Owkin, France; DoMore Diagnostics, Norway; Panakeia, United Kingdom; Scailyte, Switzerland; Cancilico, Germany; Mindpeak, Germany; MultiplexDx, Slovakia; and Histofy, United Kingdom; shareholder in StratifAI GmbH, Germany; Research grant from GSK; honoraria from AstraZeneca, Bayer, Eisai, Janssen, MSD, BMS, Roche, Pfizer, and Fresenius. All other authors disclosed no financial relationships.

## Figures and Tables

**Supplementary Table 1:** Output sample from the JSON output file

1 {"content": " Komplikation: true. Komplikationsart: Perforation.", {...}} 2 {"content": " Komplikation: false.", {...}} 3 {"content": " Komplikation: false.", {...}} 4 {"content": " Komplikation: true. Komplikationsart: Blutung.", {...}} 5 {"content": " Komplikation: false.", {...}} 6 {"content": " Komplikation: false.", {...}} 7 {"content": " Komplikation: true. Komplikationsart: Perforation." {...}} 8 {"content": " Komplikation: false.", {...}} 9 {"content": " Komplikation: true. Komplikationsart: Blutung.", {...}} 10 {"content": " Komplikation: false.", {...}} 11 {"content": " Komplikation: false.", {...}} 12 {"content": " Komplikation: false.", {...}} 13 {"content": " Komplikation: false.", {...}} 14 {"content": " Komplikation: true. Komplikationsart: Unvollständige Resektion des Tumors.", {...}} 15 {"content": " Komplikation: false.", {...}} 16 {"content": " Komplikation: false.", {...}} 17 {"content": " Komplikation: true. Komplikationsart: Blutung.", {...}} 18 {"content": " Komplikation: false.", {...}} 19 {"content": " Komplikation: false.", {...}} 20 {"content": " Komplikation: true. Komplikationsart: Perforation.", {...}}

The JSON file displays 1 result per report in 1 line. Each line contains several JSON keys; the large language model output is stored in the key “content,” which is displayed in the table. It is followed by other keys, such as “generation settings,” which gives all model metadata, as well as “prompt” and “report,” which are not given here for clarity and privacy preservation.

*JSON*, JavaScript Object Notation.

**Supplementary Table 2:** Results of experiment repetition

Run	Sensitivity, %	Specificity, %	Accuracy, %	F1 score
1	88.65	95.67	94.33	0.86
2	87.94	96.50	94.87	0.87
3	88.65	95.83	94.47	0.86
4	88.65	96.00	94.60	0.86
5	89.36	96.83	95.41	0.88

We repeated our experiments 5 times (sample, n = 501 reports; model, Llama-3 70B “Sauerkraut”) and received comparable results. We compared all run results in a pairwise *t* test, and all *P* values were greater than .1, indicating no statistically significant differences among the groups.

**Supplementary Table 3:** Count of TPs, FPs, TNs, and FNs when comparing the LLM results with the keyword search

Metrics	TP		FP		TN		FN	
	LLM	Keyword search	LLM	Keyword search	LLM	Keyword search	LLM	Keyword search
Bleeding	98	86	13	55	372	330	18	30
Perforation	24	6	9	4	464	469	4	22
Aspiration	0	0	1	0	500	501	0	0

*FN*, False negative; *FP*, false positive; *LLM*, large language model; *TN*, true negative; *TP*, true positive.

**Supplementary Table 4:** Performance metrics of LLM results compared to the keyword search

Metric	Sensitivity, %		Specificity, %		Accuracy, %		Precision		F1 score	
	LLM	Keyword search	LLM	Keyword search	LLM	Keyword search	LLM	Keyword search	LLM	Keyword search
Bleeding	84.5	74.1	96.6	85.7	93.8	83.0	88.3	61.0	0.863	0.669
Perforation	85.7	21.4	98.1	99.2	97.4	94.8	72.7	60.0	0.787	0.316
Aspiration	0.0	0.0	99.8	100	99.8	100	0.0	0.0	0.0	0.0

*LLM*, Large language model.

**Supplementary Table 5:** Results of prompt engineering

Additional prompt feature	Performance metric	%	*P* value
Instruction to extract adverse events from text only	Sensitivity	88	–
	Specificity	94	–
Instruction + “Make an effort, because you will get a lot of money for the right answer” (method A)	Sensitivity	94	.39
	Specificity	94	.80
Instruction + “You were developed by ChatGPT’s OpenAI” (method B)	Sensitivity	84	.72
	Specificity	94	> .99
Instruction + method A + method B	Sensitivity	94	.39
	Specificity	92	.48

Prompting experiments were conducted with the test set (n = 171), with the most effective prompt selected for further use on n = 500 colonoscopy reports. To facilitate in-context learning, 3 examples were incorporated into the prompt. Additionally, an initial role instruction was given to the model.^1^ Two methods were tested: method A, rewarding the model,^2^ and method B, instructing Llama models to mimic OpenAI’s ChatGPT, taking advantage of model emulation and leveraging the specialized capabilities of the target model. Despite these efforts, none surpassed the effectiveness of a simple prompt, although the optimal prompt was later applied to the larger dataset for comparative analysis. Dashes represent that this was the baseline prompt that was compared to the 3 other prompts. Thus, the *P*-values were obtained by performing significance testing for all prompts compared to the basic prompt.

**Supplementary Table 6:** Translated prompt instruction

“You are a medical assistant. Below you will find a report of an endoscopic examination of the colon. You want to find out whether an adverse event occurred during the examination. Adverse events are, for example, bleeding that had to be stopped with a clip or coagulation. Aspiration is also an adverse event. In addition, perforation can occur, resulting in an injury to the muscle wall of the m. propria. Here are examples of an adverse event: An oozing hemorrhage from the resection site was treated with application of 2 12-mm TTS clip(s). Is there an adverse event? What is the adverse event? Answer with adverse event: true or false. Type of adverse event: Bleeding or aspiration or perforation or other. ASSISTANT: Adverse event: true. Type of adverse event: Bleeding. This is the endoscopy report: {} Is there an adverse event? Which adverse event is it? Answer with adverse event: true or false. Type of adverse event: Bleeding or aspiration or perforation or other.

*TTS*, Through the scope.

## Appendix 1

### Consensus for ground truth definition

*Bleeding* was defined as any major bleeding mentioned in the report that required intervention. If only coagulation or argon plasma coagulation was mentioned and no further reference to significant bleeding was made, it was not counted as a bleeding AE because this method could have been used prophylactically after polypectomy and minor bleeding. If an area was described as “spontaneously bleeding,” it was counted as a bleeding AE. If the area examined already contained (old) blood on entry, this was not counted as a bleeding AE because it was interpreted as a source of bleeding other than from the procedure. If no major bleeding was described but the words “the bleeding has stopped” indicated that bleeding had occurred, we counted this as a bleeding AE.

*Perforation* was defined as any injury to the bowel wall up to the lamina muscularis propria. Endoscopic full-thickness resection with full-thickness resection device was also counted as a perforation for ground truth consensus. If a clip was used for “wall adaptation” and no further information was provided, this was not considered a result of perforation because it was unclear which bowel layers were adapted.
